# Does Current General Mental Health Status Relate to Current Smoking Status in Pregnant Women?

**DOI:** 10.1155/2019/7801465

**Published:** 2019-05-02

**Authors:** Diane Liu, Emily Younger, Stacy Baker, Stephanie Touch, Tamara Willmoth, Jessica L. Hartos

**Affiliations:** Department of Physician Assistant Studies, University of North Texas Health Science Center, USA

## Abstract

**Purpose:**

Research shows that smoking during pregnancy is related to mental health diagnoses. The purpose of this study was to assess whether current general mental health status is related to current smoking status in pregnant women after controlling for other factors related to both mental health and tobacco use during pregnancy.

**Methods:**

This cross-sectional analysis used 2017 Behavioral Risk Factor Surveillance Survey (BRFSS) data for 621 pregnant women aged 18-38 from Florida (N=136), Kansas (N=116), Minnesota (N=105), Nebraska (N=90), New York (N=78), and Utah (N=96). Multiple logistic regression analysis was used to assess the relationship between current mental health status and current tobacco use, while controlling for state, depression diagnosis, routine checkup, healthcare plan, age, marital status, ethnicity/race, education level, income level, and employment status.

**Results:**

Overall, very few participants reported current smoking (6%) and about one-third reported low or moderate mental health status in the past 30 days. Adjusted results indicated that those who reported high mental health status were about 3 times less likely (OR=0.29, 95% CI=0.09, 0.88) to report current smoking status compared to those who reported low mental health status.

**Conclusions:**

Overall, current mental health status was highly related to current smoking status in pregnant women. Clinicians in obstetrics may expect a very low proportion of pregnant women to report smoking and up to one-third to report low or moderate current general mental health status. Given that current mental health issues and current tobacco use may harm both mother and child, be highly related in pregnant women, and change throughout the pregnancy, pregnant women should be screened automatically for both at each visit.

## 1. Introduction

In the United States, up to 15% of pregnant women may report smoking [1–8]. This is a major public health concern as abundant research findings indicate that tobacco use during pregnancy is related to adverse, yet preventable, health effects for both of the mother and her baby. Such effects include reduced fertility, increased complications during pregnancy, low birth weight, birth defects, and long-term negative impacts on children's cognitive and behavioral functioning [1, 3–5]. Tobacco use during pregnancy has been linked to several demographic, socioeconomic (SES), and health-related factors. For example, pregnant women who smoke are more likely to be of younger ages, non-Hispanic, and not married [1–3, 5, 6] and of lower SES status, including lower income and education levels [1–3, 5, 7]. Moreover, tobacco use during pregnancy is more common among pregnant women who lack prenatal and antenatal care [4, 6] and health insurance [3, 5].

Mental health is also an important issue during pregnancy with as many as 30% of pregnant females experiencing depression [7–11]. This is also of public health concern as mental health issues can have similar negative health effects for both of the mother and her baby, including increased complications during pregnancy, low birth weight, birth defects, and long-term negative impacts on children's cognitive and behavioral functioning [9]. In addition, similar factors are related to depression in pregnant women including marital status, SES, and less prenatal and perinatal care [7, 9, 11]. Furthermore, depression during pregnancy has been related to a history of mental illness [9].

Mental health is also linked to tobacco use during pregnancy [9] as there were up to 40% of pregnant women with mental health diagnoses reporting tobacco use [7, 8]. However, most studies addressing smoking and mental health among pregnant women focus on mental health diagnoses and not general mental health during pregnancy [7–12]. Unfortunately, providers cannot necessarily conduct surveys or interviews for formal mental health diagnoses to screen for mental health issues during pregnancy visits, but they can ask about general mental health and well-being to determine whether further intervention may be needed. In addition, both smoking and mental health issues during pregnancy share similar relations with health-related, demographic, and SES factors. Therefore, the purpose of this study was to assess whether general mental health status is related to smoking status in pregnant women after controlling for factors related to both mental health and tobacco use during pregnancy.

## 2. Materials and Methods

### 2.1. Design

This cross-sectional analysis used 2017 Behavioral Risk Factor Surveillance Survey (BRFSS) conducted by the Center for Disease Control and Prevention (CDC) [13]. BRFSS is a national telephone survey system that collects data concerning health-related risk behaviors, chronic health conditions, and preventative services from over 400,000 individuals aged 18 years or older annually. BRFSS data is collected by random-digit dialing over landline and cellular telephones in the 50 US states and the District of Columbia. The CDC compiles all BRFSS data and provides deidentified data to researchers to conduct secondary data analyses. This study was given exempt status by the Institutional Review Board of the University of North Texas Health Science Center.

### 2.2. Sample

This sample included 621 pregnant women aged 18-38 from Florida (N=136), Kansas (N=116), Minnesota (N=105), Nebraska (N=90), New York (N=78), and Utah (N=96) who had data for mental health status and smoking status. These states were selected based on the highest numbers of pregnant women participants and prevalence of smoking according to the 2016 prevalence maps [14].

### 2.3. Data

The 2017 BRFSS data was used for all variables [15]. The outcome, tobacco use, was categorized “yes” or “no” to current smoker (i.e., combining responses to smoked at least 100 cigarettes in lifetime and now smokes “some days” or “every day”). The factor of interest, mental health status, was measured in BRFSS as “Now thinking about your mental health, which includes stress, depression, and problems with emotions, for how many days in the past 30 days was your mental health not good,” and responses were categorized “low (0 days)” “moderate (1-13 days),” and “high (14+ days).” We reversed this variable to reflect “good” mental health as “low (16 good days or less),” “moderate (17-29 good days),” and “high (30 good days)” mental health status.

Control variables included state, depression diagnosis, routine checkup, healthcare plan, age, marital status, ethnicity/race, education level, and income level. Previous BRFSS data shows that tobacco use varies by state [2]. Depression diagnosis was measured as “yes” or “no” to ever being diagnosed with depression or dysthymia. Routine checkup was measured as “yes” or “no” to visiting a doctor in the past 12 months. Healthcare plan was measured as “yes” or “no” to having any kind of private or public healthcare coverage. Age categories included “18-30” and “31-38.” Marital status was measured as “married” or “other.” Because the majority of respondents were of white race, ethnicity/race was measured as “white” or “other.” Education level was measured as “graduated high school or less” or “some college/technical school or more.” Income level was measured as “less than $25,000” or “$25,000 or more.” Employment status was measured “employed” or “other.” All variables and categories are shown in Table 1.

### 2.4. Analysis

Frequency distributions were generated for each variable by state to describe participant characteristics and to evaluate the distribution of variables. Multiple logistic regression analysis using combined state data was conducted to assess the relationship between tobacco use and mental health status while controlling for state and health-related, demographic, and socioeconomic factors. Any observations with missing data for any variables were excluded from the adjusted analysis. All analyses were conducted in Stata 15.1 (Copyright 1985-2017 StataCorp LLC).

## 3. Results

### 3.1. Descriptive Statistics

For pregnant females aged 18-38, very few reported current smoking (n=40, 6%). Other participant characteristics are shown in Table 1. For mental health, about one-third reported low or moderate mental health in the past 30 days. For health-related variables, about two-thirds or more reported no prior depression diagnosis, having a routine checkup within the past year, and having a healthcare plan. For demographic factors, about half reported being between the ages of 30 and 38 years old, and about two-thirds reported being married and of white race. For socioeconomic status, the majority of women reported some college/technical school or more, an income greater than or equal to $25,000, and being employed.

### 3.2. Unadjusted and Adjusted Statistics

As shown in Table 1, the results of simple logistic regression analysis for pregnant females aged 18-38 showed that mental health status was highly related to smoking status. Compared to those who reported low mental health status, pregnant women who reported moderate mental health status were about 3 times less likely to report current smoking status, and those who reported high mental health status were about 5.5 times less likely to report current smoking status. In addition, most control variables were significantly related to smoking status including depression diagnosis, routine checkup, marital status, ethnicity/race, education level, and income level. However, only three independent variables remained significant in adjusted analysis.

Also shown in Table 1, the results of multiple logistic regression analysis indicated that, after controlling for all other variables in the model, tobacco use during pregnancy was related to mental health status. Participants who reported high mental health were about 3.4 times less likely to report tobacco use during pregnancy when compared to those who reported low mental health status. In addition, demographic variables with independent contributions to tobacco use during pregnancy included “other” marital status and “white” ethnicity/race.

## 4. Discussion

The purpose of this study was to assess whether current mental health status was related to current smoking status in pregnant women after controlling for other factors related to both tobacco use and mental health during pregnancy. In this study, very few pregnant women reported current smoking (6%) and about one-third reported low or moderate mental health status in the past 30 days. The results of multiple logistic regression indicated that, after controlling for other variables related to both smoking and mental health during pregnancy, current general mental health was significantly related to tobacco use for pregnant women as participants who reported high mental health status were about 3 times less likely to report current smoking status. These results are similar to previous results for the relationship between mental health diagnoses and tobacco use during pregnancy [7, 8, 11]. However, this study differs from previous research because we assessed the past 30-day general mental health status in pregnant women and not specific mental health disorders using structured or diagnostic criteria [7–11]. Such one-item general mental health measures that could be easily incorporated into doctor visits may be useful in determining whether patients need further screening for mental health issues. Future research should assess the validity and reliability of such procedures in practice.

### 4.1. Limitations

The use of BRFSS data allowed access to a population-based samples of currently pregnant women; however, pregnant participants by state in BRFSS 2017 were relatively low (our n's = 78 to 136). In addition, there were a small proportion of pregnant women who reported current smoking status, which may be an underrepresentation as pregnant women may be hesitant to respond truthfully to questions regarding smoking status [2]. Furthermore, although BRFSS provides current data for mental health status, smoking status, and pregnancy status, other potentially relevant information was not available, such as the severity and management of one's mental health issues or other pregnancy-related factors such as hormone levels that may influence mental health status. Moreover, BRFSS data only had measures for a single time point during pregnancy whereas both smoking and mental health issues may fluctuate as pregnancy progresses [9, 10]. Finally, direction of causality could not be determined in this study and research is mixed on whether mental health issues contribute to smoking or the other way around [16].

### 4.2. Conclusion

As this study used population-based data, the results may generalize to pregnant women aged 18-38 in obstetrics. As such, there may be a very low proportion of pregnant women who report smoking and a moderate proportion who report low or moderate current general mental health status. However, because mental health issues and tobacco use may harm both mother and child, be highly related, and fluctuate during pregnancy, clinical health providers should always screen pregnant women for both tobacco use and general mental health status at each visit. They should also educate patients on the health risks of smoking and importance of good mental health before, during, and after pregnancy and provide mental health referrals and/or smoking cessation resources as needed.

## Figures and Tables

**Table 1:** Participant characteristics, unadjusted results, and adjusted results.

Predicting tobacco use (Yes vs. no)	Descriptive statistics ^a^		Unadjusted statistics ^b^			Adjusted statistics ^b^		
	N=621		*OR*	95% CI		*OR*	95% CI	
	N	%		Low	High		Low	High
Mental health status	621	100						
Low	63	10	ref	-	-	ref	-	-
Moderate	154	25	*0.35*	0.14	0.87	0.42	0.14	1.28
High	404	65	*0.18*	0.08	0.42	*0.29*	0.09	0.88

Depression diagnosis	621	100						
No	494	80	ref	-	-	ref	-	-
Yes	127	20	*4.93*	2.46	9.89	2.33	0.86	6.30

Routine checkup	605	97						
More than one year	177	29	ref	-	-	ref	-	-
Within the past year	428	71	*0.49*	0.25	0.97	0.81	0.35	1.86

Healthcare plan	621	100						
No	77	12	ref	-	-	ref	-	-
Yes	544	88	0.59	0.26	1.35	0.70	0.25	1.99

Age	621	100						
18 – 29	332	53	ref	-	-	ref	-	-
30 – 38	289	47	0.83	0.43	1.60	1.37	0.58	3.21

Marital status	609	98						
Other	211	34	ref	-	-	ref	-	-
Married	409	66	*0.16*	0.08	0.34	*0.20*	0.08	0.53

Ethnicity/race	611	98						
Other	198	32	ref	-	-	ref	-	-
White	413	68	*2.58*	1.09	6.11	*3.65*	1.17	11.38

Education level	618	100						
High school or less	167	27	ref	-	-	ref	-	-
Some college or more	451	73	*2.63*	1.25	5.55	2.28	0.81	6.41

Income level	541	87						
Less than $25,000	162	30	ref	-	-	ref	-	-
$25,000 or more	379	70	*3.29*	1.62	6.66	1.56	0.58	4.16

Employment status	612	99						
Other	239	39	ref	-	-	ref	-	-
Employed	373	61	0.65	0.33	1.26	1.04	0.44	2.48

^a^ Variable %s are based on the sample total N; category %s are based on the variable total N.

^b^ All models are adjusted for state.

*Note. OR =* odds ratio; 95% CI = 95% confidence intervals; ref=referent group; italic indicates significance (*OR*s with 95% CI that do not include 1.00 are significant).

## Data Availability

BRFSS data is freely available from Centers for Disease Control and Prevention (CDC) 2017 BRFSS survey data and documentation 2018 https://www.cdc.gov/brfss/annual_data/annual_2017.html.
